# Applicability of a single‐use bioreactor compared to a glass bioreactor for the fermentation of filamentous fungi and evaluation of the reproducibility of growth in pellet form

**DOI:** 10.1002/elsc.202000069

**Published:** 2021-02-25

**Authors:** Winda Soerjawinata, Konstantin Schlegel, Natalie Fuchs, Anja Schüffler, Tanja Schirmeister, Roland Ulber, Percy Kampeis

**Affiliations:** ^1^ Institute for Biotechnical Process Design Trier University of Applied Sciences, Environmental Campus Birkenfeld Hoppstädten‐Weiersbach Germany; ^2^ Institute of Pharmaceutical and Biomedical Sciences Johannes Gutenberg University Mainz Mainz Germany; ^3^ Institut für Biotechnologie und Wirkstoff‐Forschung gGmbH (IBWF) Mainz Germany; ^4^ Institute of Bioprocess Engineering Technical University Kaiserslautern Kaiserslautern Germany

**Keywords:** fermentation, pellets, *Penicillium* sp, protease inhibitor, single‐use bioreactor

## Abstract

The implementation of single‐use technologies offers several major advantages, e.g. prevention of cross‐contamination, especially when spore‐forming microorganisms are present. This study investigated the application of a single‐use bioreactor in batch fermentation of filamentous fungus *Penicillium* sp. (IBWF 040‐09) from the Institute of Biotechnology and Drug Research (IBWF), which is capable of intracellular production of a protease inhibitor against parasitic proteases as a secondary metabolite. Several modifications to the SU bioreactor were suggested in this study to allow the fermentation in which the fungus forms pellets. Simultaneously, fermentations in conventional glass bioreactor were also conducted as reference. Although there are significant differences in the construction material and gassing system, the similarity of the two types of bioreactors in terms of fungal metabolic activity and the reproducibility of fermentations could be demonstrated using statistic methods. Under the selected cultivation conditions, growth rate, yield coefficient, substrate uptake rate, and formation of intracellular protease‐inhibiting substance in the single‐use bioreactor were similar to those in the glass bioreactor.

**Abbreviations**
BDM: bio dry mass
BLAST: Basic Local Alignment Search Tool
DO: dissolved oxygen
Glu: glucose
HPLC: high performance liquid chromatography
IBWF: *Institut für Biotechnologie und Wirkstoff‐Forschung gGmbH*
ITS: Internal Transcribed Spacer
Mal: maltose
SU: single‐use
Suc: sucrose
YMG: yeast extract, malt extract, and glucose

## 1 INTRODUCTION

The application of and the interest in single‐use (SU) technologies in biopharmaceutical industries have been growing since the first single‐use wave‐induced agitation bioreactor was launched in 1998 [1]. The first single‐use stirred‐tank bioreactor appeared in 2004 [2]. The implementation of single‐use technologies offers several major advantages, such as the reduced workload for preparation, cleaning, sterilization, and validation, reduced water and energy consumption, reduced risks of cross‐contamination, and flexibility of the production processes [1, 2, 3, 4]. In the biopharmaceutical industries, cross‐contamination is one of the important aspects that needs to be considered, particularly when the microorganisms involved in the process are spore‐forming ones. Spore contaminations from previous processes might be caused by improper cleaning and sterilization processes of the bioreactor. The use of SU bioreactors can eliminate this because such bioreactors will be disposed directly after the collection of the fermentation broth. In addition, spores formed in multi‐purpose process lines can cause problems in the clean room if the process equipment has not been adequately cleaned due to their easy spread through air circulation.

A commonly used stirred SU bioreactor was the UniVessel^®^ SU bioreactor from Sartorius Stedim Biotech GmbH, which is equipped with a two‐stage impeller. The physical parameters of this bioreactor type such as mixing time, power input, and mechanical stress, have been extensively investigated in several previous studies [5, 6, 7, 8]. Furthermore, Schirmaier et al. (2014) [8] and Zanghi et al. (2017) [9] have shown the potential of UniVessel^®^ SU for stem cell production and Chinese Hamster Ovary (CHO) cell lines cultivation, respectively. Up until now, applications of SU bioreactors are generally still limited for cell culture [10, 11], plant cell culture [12], microalgae [13], bacteria [14, 15], and yeast [16]. To the best of our knowledge, there is no published report yet on the application of SU bioreactors, especially that of the UniVessel^®^ SU for fungal fermentation. However, with the advantages described above, SU bioreactors have the potential to be used in fungal fermentations.

**PRACTICAL APPLICATION**

This study investigated the potential application of single‐use bioreactors in pellet‐forming fungi fermentation of *Penicillium* sp. (IBWF 040‐09). With some modifications on the single‐use bioreactor provided in this manuscript, this filamentous fungus showed similar behavior in growth rate, yield coefficient, and substrate uptake rate, compared to a glass bioreactor as reference. In addition, the reproducibility of the fermentations in glass and single‐use bioreactors could be shown under the cultivation procedure described. Moreover, a protease inhibiting substance was also produced by the fungus using this modified set‐up. This study offers the opportunity for biopharmaceutical industries to prevent cross‐contamination within the fermentation system using single‐use bioreactors, particularly when spore‐forming microorganisms are involved.

Fungi are usually cultivated in conventional stirred bioreactors. Based on the studies reporting fermentations of various *Penicillium* in conventional stirred bioreactors, the biomass yield coefficient ranges from 0.45 to 0.61 g_X_/g_S_ [17, 18, 19, 20, 21]. In fungal fermentation, many factors must be considered, such as mass transfer, mixing process, and shear stress. Depending on the strain and process parameters, such as agitation rate, fungi can be fermented in two different morphologies: As pellets or as free mycelia [22, 23]. Higher agitation rate leads to higher shear stress, which reduces pellet size or leads to free mycelium, increasing the viscosity of the fermentation broth [22, 24]. This fact is supported by the study of Veiter et al. (2020) [25], in which power input correlates with pellet size as well as pellet compactness.

*Penicillium* sp. (IBWF 040‐09) used in this study was found to produce a protease inhibitor against parasitic proteases, such as rhodesain, the major cysteine protease from *Trypanosoma brucei rhodesiense*. This protease inhibitor can be extracted from the biomass and has the potential to be used to treat the parasitic disease *Human African Trypanosomiasis* (African sleeping sickness) that is transmitted by the bite of the tsetse fly [26]. Based on the preliminary studies, a protease inhibitor as a secondary metabolite was only produced intracellularly. The growth of fungi in the form of free mycelia produces some negative consequences in the fermentations. Sensors and internals in the bioreactors, such as the dissolved oxygen sensor, become much more overgrown and the viscosity of the fermentation broth is very high, making homogeneous mixing difficult. Thus, fungal growth in pellet form was preferred in this study. The cultivation conditions, such as agitation intensity, gas supply, spore concentration, were optimized in preliminary studies to allow fermentation leading to pellet formation.

The first objective of this study was to examine the feasibility of UniVessel^®^ SU bioreactors from Sartorius Stedim Biotech GmbH in batch fermentations of filamentous fungi *Penicillium* sp. (IBWF 040‐09). Since the used bioreactor type was initially designed for cell culture cultivation, several modifications to the bioreactor were required to enable and further optimize the fermentation process with the used fungus.

A classical glass bioreactor with approximately the same geometry served as a reference system under the same cultivation conditions. A reliable statement was to be made as to whether fungal fermentation in the SU bioreactor delivers the same results in terms of biomass growth, yield coefficient, substrate uptake rate and product formation as in the glass bioreactor. Therefore, as a second focus, it had to be investigated whether the fermentation in the glass bioreactor is reproducible at all under the selected process conditions and whether it is suitable as a reference system. Since both the data of the fermentations in SU bioreactors and the data of the fermentations in glass bioreactors are subject to measurement errors, statistical methods are necessary to verify whether the same metabolic activity of the fungus is present in the SU bioreactor as in the glass bioreactor — despite the completely different construction material and the different gassing system. The polycarbonate used as the construction material could possibly lead to a different adhesion of the fungus to the reactor wall, and the different gassing system could lead to a reduced space‐time yield of the bioreactor.

## 2 MATERIALS AND METHODS

### 2.1 Glass bioreactor

As glass bioreactor an UniVessel^®^ Glass DW 2 L from Sartorius Stedim Biotech GmbH with working volume of 0.4‐2 L was used. The diameter and height of the vessel were 130 and 240 mm, respectively. The bioreactor was equipped with 2 pieces of 3‐blade segment impeller with angle of 30 degrees. The distance between impellers was 55 mm and the lower impeller was located 27 mm from the bottom of the stirrer shaft. The fermentation media was aerated by sterile‐filtered air through the ring sparger with 14 holes. An exhaust‐gas cooler was used in order to condensate the vapor emerged during fermentation process. The exhaust gas left the bioreactor through an exhaust‐gas sterile filter (Midisart 0.2 µm, Sartorius Stedim Biotech GmbH). Between the exhaust‐gas cooler and the exhaust‐gas filter, a wash‐bottle was installed which was filled with edible oil in order to separate spores that pass the exhaust‐gas cooler. Without this, the spores will block the exhaust‐gas filter very quickly. In addition, a reserve filter was installed, which can be used in place of the exhaust‐gas filter if it is blocked, without having to interrupt the gassing or fermentation as a whole. The pH value of the fermentation broth was measured using a standard pH sensor (EasyFerm Plus PHI 325, Hamilton Company). The pH sensor was calibrated using pH buffers (pH = 4 and 7) prior to the sterilization of the bioreactor in an autoclave. An optical dissolved oxygen sensor (VisiFerm DO 325, Hamilton Company) was used to perform online‐measurement of the dissolved oxygen (DO). The DO sensor was calibrated after sterilization. Atmospheric air and nitrogen were used for the two‐point calibration of the DO sensor.

### 2.2 Single‐use bioreactor

UniVessel^®^ Single‐Use Bioreactor 2 L (SU bioreactor) from Sartorius Stedim Biotech GmbH was used to carry out fermentations in disposable bioreactors. This SU bioreactor has a working volume of 0.6‐2 L and is made out of polycarbonate. The inner diameter is 130 mm and the inner height 242 mm. It is equipped with pH und DO sensor patches which enable to perform non‐invasive on‐line measurements during cultivation. Two 3‐blade segment impellers with angle of 30 degrees are mounted on the impeller shaft for mixing with low shear stress. The distance between the impellers was 70.2 mm and the lower impeller was 47.3 mm from the bottom of the stirrer shaft. This SU bioreactor also offers two approaches for aeration: overlay and L‐sparger [4]. During the fermentation, the bioreactor was aerated by sterile filtered air through the L‐type sparger with 16 holes (0.5 mm diameter). Both inlet air and exhaust gas were filtered with sterilizing‐grade air filters (Midisart 0.2 µm, Sartorius Stedim Biotech GmbH) similar to the fermentations in the glass bioreactor.

### 2.3 Strain and inoculum preparation

The microorganism used in this study was *Penicillium* sp. (IBWF 040‐09) which was isolated from a soil sample collected in 2009 in Germany. By morphology the strain was identified as *Penicillium* but to define it more precisely the Internal Transcribed Spacer (ITS) sequence was analyzed using Basic Local Alignment Search Tool (BLAST) which showed 99.29 to 99.82% homologies with *P. antarcticum*, *P. coralligerum*, and *P. atrovenetum* (see Supporting Information for sequence and identity). Strain preparation was carried out on agar plates containing yeast and malt extract with glucose (YMG). YMG agar consisted of 9 g/L glucose, 10 g/L malt extract, 4 g/L yeast extract, 20 g/L agar, and was adjusted to pH = 5.5 using 1 M HCl before autoclaving. The agar plates were incubated at 22 °C in an incubator (INCU‐Line, IL 23R, VWR International LLC.) for 4 weeks before fermentation. To preserve the fungi, a streak plate method from 2‐week‐old spore plates to new agar plates was carried out every week. In order to prepare the inoculum for the fermentation, spores were washed out from an agar plate using sterile 0.9% NaCl solution containing 10 µL/L Triton X‐100. Triton X‐100 was used to reduce the surface tension since the spores are hydrophobic. The spores were afterwards counted using hemocytometer (Neubauer‐improved, Paul Marienfeld GmbH & Co. KG). For every fermentation in the present study, the spore concentration required for inoculum was 4.41·10^5^ spores per liter fermentation medium.

### 2.4 Fermentation conditions

The fermentation medium used in this study was YMG medium, with similar composition as YMG agar written in Section 2.3, but without agar. A volume of 2 L medium was used in all experiments. As antifoam agent 25 µL polypropylene glycol per liter medium was added before the sterilization. For the fermentation in the glass bioreactor, the medium was autoclaved in the bioreactor at 121 °C for 15 min. In the case of SU bioreactor, the bioreactor was already sterile and the medium was autoclaved separately at 121 °C for 15 min. The transfer of medium into the SU bioreactor was performed in a sterile workbench.

The following fermentation conditions were valid for all fermentations in the glass bioreactor as well as for all fermentations in the SU bioreactors. The batch fermentation of *Penicillium* sp. (IBWF 040‐09) was conducted at 22 °C, which the fungus prefers according to results of preliminary experiments. Before the fermentation was started, sterile‐filtered Pluronic F68 with an end concentration of 0.2% (v/v) was added to the medium to reduce the shear stress [27]. In the beginning of the fermentation process, the aeration through the sparger was set constant at 1 L/min (refers to 0.5 vvm). The controller of the aeration was started as soon as DO saturation reached a value below 30%. In order to maintain the minimal DO saturation at 30%, the volume flow of the atmospheric air could be raised to 2.25 L/min. If this was not sufficient to feed the fungi, pure oxygen up to 0.25 L/min could be provided additionally. The pH value during the fermentation was only measured but not controlled. The stirrer speed remained constant at 350 rpm. Since the stirrer geometries were identical in both cases (ø 54 mm), the tip speed of the stirrer was also the same (0.99 m/s). The batch fermentation was ended when the aeration rate decreased and went back to 1 L/min. During the fermentation, samples were taken regularly for further analysis of bio dry mass and substrate concentration (glucose and maltose).

### 2.5 Bio dry mass determination

Bio dry mass (BDM) of the cells, expressed in grams of dry weight per liter of culture medium (g/L), was determined gravimetrically. Samples (5 mL) that were collected during the fermentations, were filtered using Büchner funnel with pre‐dried and pre‐weighted 0.45 µm membrane filter (ø 47 mm, Cellulose Acetate Filter, Sartorius Stedim GmbH). The pellets were washed afterwards using distilled water. The filter cakes were put at 80 °C in an oven (UT12P, Thermo Electron LED GmbH) until they reached constant weight.

### 2.6 Substrate determination

Two carbon sources were determined in this study, glucose and maltose. The filtrate from BDM analysis was used for substrate analysis. The substrate analysis was carried out offline via High Performance Liquid Chromatography (HPLC) using a ligand exchange column (Reprogel Ca^2+^, 9 µm, 300 × 8 mm, Dr. Maisch GmbH) with ultrapure water as mobile phase. The flowrate was adjusted to 0.6 mL/min. The column was tempered to 80 °C in a column oven (K7, Techlab GmbH). The HPLC system used was an instrument combination SCL‐10AVP/LC‐10AD with a refractive index detector RID‐10A from Shimadzu Europa GmbH. Prior to the injection into HPLC, the samples were filtered using 0.2 µm PES membrane syringe filter (Filtropur S 0.2, Sarstedt AG & Co. KG). The quantitative analysis was carried out by determining the peak areas using the Clarity 7.1 software form DataApex Company. The retention time of maltose and glucose in this experimental set‐up were 8.9 and 10.5 min, respectively.

### 2.7 Extraction and chromatographic separation of protease inhibitor

Preliminary studies were carried out to determine the presence of protease inhibitor as intracellular or extracellular product. For this purpose, extracts were obtained from the cell mass and the fermentation supernatant. Afterwards defined fractions of these extracts were analyzed in a bioassay (see Section 2.8). These studies proved that the active substance was found in mycelium extract. An activity could not be detected in the supernatant.

The extraction process of biomass was started by filtering 50 mL samples using a filtration glass funnel (Sartorius Glass Funnel, Sartorius Stedim GmbH) with 0.45 µm membrane filter (ø 47 mm, Cellulose Acetate Filter, Sartorius Stedim GmbH). Since the active substance is a secondary metabolite, the samples were taken at the time when the substrate was completely consumed, which means at the end of the fermentations.

The filter cake (mycelium) was washed using distilled water. The washed mycelium was frozen in the ultra‐low freezer (B 35‐85, FRYKA‐Kältetechnik GmbH) and dried in a freeze dryer (ALPHA 1–2 LD plus, Martin Christ GmbH). The freeze‐dried cells were mixed with 50 mL extraction solvent consisting of methanol and acetone (MeOH:(CH_3_)_2_CO = 1:1) and disrupted in an ice bath for 30 min using a homogenizer (Polytron PT 1600 E with dispersing aggregate PT‐DA 12/2 EC‐E157, Kinematica AG). After extraction, the cell mass was separated in a two‐step filtration. For the pre‐filtration a vacuum pump and a Büchner funnel with a glass‐fiber round filter (ø 50 mm, Schleicher & Schuell GmbH) were used. In a second step, the pre‐filtrate was filled into a 50 mL syringe and sterile‐filtered through a 0.45 µm PTFE membrane syringe filter (Minisart^®^ Plus, Sartorius Stedim GmbH). Then, the extract was concentrated using a rotary evaporator (Laborota 4000 efficient, Heidolph Instruments GmbH & Co. KG). Spinning speed was adjusted to 120 rpm. At a pressure of 340 mbar_abs_ and an oil bath temperature of 42 °C, the acetone evaporated first. After approximately half of the extraction solvent had evaporated, the pressure was gradually adjusted to 180 mbar_abs_ and the oil bath temperature to 45 °C due to the higher boiling temperature of methanol. The extract was concentrated until a residue of about 0.5‐1 mL remained. For 50 mL sample this resulted in a 50 to 100‐fold concentration.

These extracts were further processed using a reversed phase 1200 Series HPLC system from Agilent Technologies Inc., with the column LiChrospher^®^ 100 RP‐18, 5 µm, LiChroCART 125‐4 and pre‐column LiChrospher^®^ 100 RP‐18, 5 µm, LiChroCART 4‐4 from Merck KGaA. The 1200 Series HPLC system consisted of the degasser G1379, the binary gradient pump G1312, the column oven G1316A and the diode array detector G1315A. The quantitative analysis and control of the HPLC was performed with software Agilent ChemStation. In order to reduce clogging problems from potential particles during HPLC, the samples were filtered again in a pretreatment step using a 0.45 µm membrane syringe filter (Chromafil^®^ PET‐45/15 MS, Macherey‐Nagel GmbH & Co. KG).

The mobile phase contained acetonitrile and a 0.1% aqueous solution of trifluoroacetic acid (TFA) with a flowrate of 1 mL/min. The gradient over the run time was as follows: 0‐20 min gradient acetonitrile from 1 to 100%, 20‐23 min isocratic acetonitrile 100%, 23‐25 min gradient acetonitrile from 100 to 1%, 25‐28 min isocratic acetonitrile 1%. The column was tempered to 40 °C in the column oven. The UV detection was performed at a wavelength of 230 nm. For small scale separations the method above was used to fractionate extracts to 96‐well‐plates with a fraction collector G1364 from Agilent Technologies Inc. Therefore, 400 µg of extract were injected and fractionated between 0 and 23 min in 0.25 min slices. After drying of the solvent, the 96‐well‐plate could be used for bioassays (see Section 2.8).

### 2.8 Protease inhibition assay

The protease rhodesain was expressed as published previously [28]. The increase of fluorescence upon cleavage of the fluorogenic substrate N‐Cbz‐Phe‐Arg 7‐amido‐4‐methylcoumarin hydrochloride (N‐Cbz‐Phe‐Arg‐AMC·HCl; Bachem Holding AG) by rhodesain was monitored (λ excitation: 365 nm, λ emission: 460 nm) by a plate reader Infinite F200 Pro from Tecan Group Ltd. The enzyme stock solution (4 mg/mL in 10 mM sodium citrate buffer, pH = 5.5) was 800‐fold diluted with an enzyme buffer (50 mM sodium acetate pH = 5.5, 5 mM EDTA, 200 mM NaCl and 2 mM DTT) and incubated for 30 min at room temperature. Assays were performed in black, flat‐bottom 96‐well microtiter plates (Greiner Bio‐One International GmbH) with a total volume of 200 µL. A volume of 180 µL assay buffer (50 mM sodium acetate, pH = 5.5, 5 mM EDTA, 200 mM NaCl and 0.005% Brij35) was added to the 96‐well‐plates, followed by 5 µL of rhodesain in enzyme buffer, followed by 10 µL DMSO with or without the fractions and finally 5 µL of N‐Cbz‐Phe‐Arg‐AMC·HCl (final substrate concentration 10 µM). After addition of the substrate, the fluorescence emission was monitored directly. Every sample was measured in two independent measurements.

### 2.9 Statistical assessment of growth rate, yield coefficient, and substrate uptake rate

The growth rate (μ, expressed in h^–1^) of the fungus was determined from the slope of the linear regression from the plot natural logarithm of bio dry mass against time (see Equation 1). The yield coefficient of biomass per substrate (Y_X/S_, expressed in g g^–1^) was calculated from the difference in bio dry mass and the difference in substrate concentration within a time period (see Equation 2). Substrate uptake rate (q_S_, expressed in g g^–1^ h^–1^) was calculated from growth rate and yield coefficient (q_S_ = μ/Y_X/S_). Yield coefficient and substrate uptake rate were calculated only for glucose as substrate because glucose was consumed by the fungus within the exponential phase, whereas maltose was consumed to a greater extent only in the stationary phase.

(1) $$\mu =\frac{\ln\left(\left[X\left(t_{2}\right)\right]\right)-\ln\left(\left[X\left(t_{1}\right)\right]\right)}{t_{2}-t_{1}}$$

(2) $$Y_{x/s}=\frac{\left[X\left(t_{2}\right)\right]-\left[X\left(t_{1}\right)\right]}{\left[S\left(t_{1}\right)\right]-\left[S\left(t_{2}\right)\right]}$$

The growth rates and yield coefficients determined within the fermentations are subject to measurement errors. In order to be able to specify a confidence interval, the data from the fermentations were statistically evaluated using a *t*‐test procedure. In case of growth rate, the value to be determined in each fermentation corresponds to the slope resulting from the linear progression. The straight line of the linear regression (see Equation 3 with estimated y‐value ${\hat{y}}_{i}$) with slope a and y‐axis intercept b can be determined using the fermentation data by applying Equations 4 and 5 (with mean values $\bar{y}$ and $\bar{x}$). The confidence interval is spanned by a straight line with a smaller slope and a straight line with a larger slope in the two‐dimensional space.

(3) $${\hat{y}}_{i}=a\cdot x_{i}+b$$

(4) $$a=\frac{\sum \left(\left(x_{i}-\bar{x}\right)\cdot \left(y_{i}-\bar{y}\right)\right)}{\sum \left({\left(x_{i}-\bar{x}\right)}^{2}\right)}$$

(5) $$b=\bar{y}-a\cdot \bar{x}$$

However, there is a problem for statistical evaluation: The positions of the straight lines differ between the individual fermentations in the x‐ and y‐directions due to different lengths of the lag phase and the exponential phase, respectively. Therefore, the individual data points ln([X(t)]);t from the fermentations cannot be used directly in a statistical analysis. To overcome this problem, the straight lines from all fermentations were shifted to the point 0;0 by subtracting the relevant mean value from all ln([X(t)]) values or from all t values at each fermentation. Then, with the entire data of all fermentations, a statistical analysis can be performed using the *t*‐test method with n‐2 degrees of freedom and 95% prediction accuracy to give a 95% confidence interval. To perform this, Equation 6 was first used to calculate the variance s^2^ of the data.

(6) $$s^{2}=\frac{1}{n-2}\cdot \frac{\sum \left({\left(y_{i}-{\hat{y}}_{i}\right)}^{2}\right)}{\sum \left({\left(x_{i}-\bar{x}\right)}^{2}\right)}$$

Then, the quantile q of a *t*‐distribution for the desired confidence interval with the prediction inaccuracy *α* = 0.05 is taken from the corresponding table (see Equation 7).

(7) $$q\left(1-\frac{\alpha }{2}\right)=q\left(1-\frac{1-0.95}{2}\right)=q\left(0.975\right)$$

From the expected value a of the slope of the straight line obtained from all fermentation data, the variance of the data and the quantile of the *t*‐distribution, a confidence interval for the growth rate μ can now be calculated using Equation 8.

(8) $$\mu =a\pm \sqrt{s^{2}}\cdot q\left(1-\frac{\alpha }{2}\right)$$

An analogous procedure with the data points Δ[X];Δ[S] was performed to determine a confidence interval for the yield coefficient. However, since no time component is included here, the straight lines did not need to be shifted to a reference point in two‐dimensional space for statistical evaluation. A calculation of the yield coefficient using the arithmetic mean and its standard deviation, what would be possible here, was not performed due to the instability of this statistical method to outliers.

In addition, the Wilcoxon‐Mann‐Whitney test, as presented in [29], was applied to the data to test whether or not the growth rates and yield coefficients were statistically significantly different between the fermentations in SU bioreactors and glass bioreactor.

## 3 RESULTS AND DISCUSSION

*Penicillium* sp. (IBWF 040‐09) was cultivated in two different bioreactors, UniVessel^®^ SU and UniVessel^®^ glass, under the cultivation conditions described in Section 2.4. Every fermentation took about 2 weeks from the production of the inoculum (spore suspension) to the determination of the BDM. In the following, the typical behavior as well as the reproducibility of the fermentation in the glass bioreactor is presented. For cultivating the filamentous fungus in the SU bioreactor under the same cultivation conditions, some modifications to the SU bioreactor were required. In order to test whether the fermentation in the two bioreactor types — despite the different construction material and the different gassing system — proceeds with the same metabolic activity of the fungus, biomass yields, biomass growth rates, yield coefficients related to glucose and substrate uptake rates were determined. Confidence intervals were calculated for the comparison of the two bioreactor types and whether or not there was a statistically significant difference.

### 3.1 Morphology of *Penicillium* sp. during fermentations

Generally, there are two options in cultivating filamentous fungi: as loose mycelia or as pellets [22, 23]. An advantage of pellet morphology is that the viscosity of the fermentation broth will remain low and therefore, mixing of the fermentation broth is unproblematic [25]. Additionally, cultivation in loose mycelia form can often result in fouling of reactor internals, such as the sensors. Mass transport in the pellets can be one of the disadvantages of pellet morphology which might also affect the productivity [30]. A comprehensive study about the correlation between morphology and mass transport in filamentous fungi pellets is also available, written by Schmideder et al. (2020) [31]. That cultivation in pellet form is less problematic for gas‐liquid mass transfer and liquid mixing due to the lower viscosity of the fermentation broth has been reported in other studies [25, 32, 33].

In the case of the protease inhibitor produced by *Penicillium* sp. (IBWF 040‐09); however, internal mass transport does not pose a serious problem since this substance is produced as a secondary metabolite only by undersupplied fungal cells. Consequently, the limited mass transport to the center of the fungal pellets should support the formation of the protease‐inhibiting substance. Therefore, it is very likely not disadvantageous if a large portion of the biomass in the pellets is already undersupplied during fermentation and not only at the end of fermentation. Due to current limitations in the isolation and quantification of the protease inhibitor by HPLC (see Section 3.8), it is not the content of the work presented here to verify whether more protease inhibitor is actually formed in fermentations with pellet‐like growth. In any case, this needs to be investigated in more detail in the future. In preliminary experiments at IBWF, it was shown that the fungus forms a satisfactory amount of the protease‐inhibiting substance when it grows in form of pellets. Due to the process‐technical advantages, a fermentation in which the fungus shows pellet‐like growth was therefore aimed for in this work.

Therefore, the submerged fermentations of *Penicillium* sp. (IBWF 040‐09) were carried out under the fermentation conditions described in Section 2.4. These were optimized in preliminary experiments to ensure growth of the fungus in pellet form and resulted in pellets with diameters between 2 and 4 mm in both bioreactor types. Higher agitation rate led to destruction of the pellets, while lower agitation rate led to problems regarding to oxygen supply and mixing. Thus, a stirring speed of 350 rpm was selected in this study, which was found to be optimal.

### 3.2 Growth of *Pencillium* sp. in glass bioreactor

**FIGURE 1:**
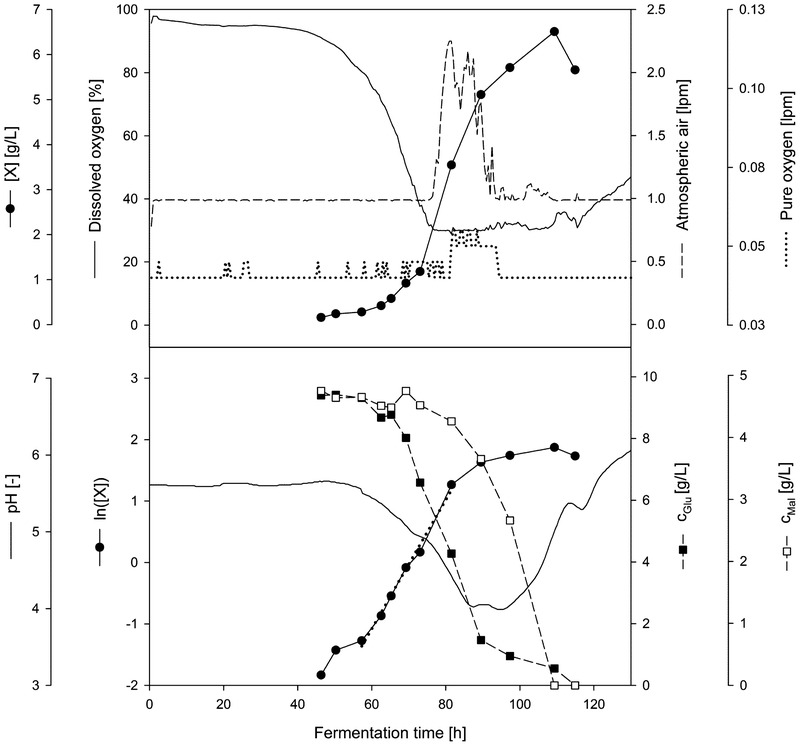
Bioreactor cultivation of *Penicillium* sp. (IBWF 040‐09) in 2 L glass bioreactor (22°C, 350 rpm, initial aeration 1 L/min) from fermentation Glass II; Top: bio dry mass, dissolved oxygen, flow rate of atmospheric air, and flow rate of pure oxygen; Bottom: natural logarithm of bio dry mass, glucose concentration, maltose concentration, and pH value; linear regression of the exponential phase is indicated by a dotted line

An example of a typical time course of submerged fermentation of *Penicillium* sp. (IBWF 040‐09) in glass bioreactor (Glass II) is shown in Figure 1. In this study, inoculations were not made with biomass but with spores. At the beginning of the fermentation process, the spores must first germinate before a measurable biomass formation can begin. A relatively long lag phase can, therefore, be expected in the process. Based on the pH course, no metabolic activity could be detected in the first 55 h. Germination and aggregation processes for pellet formation took place during this lag phase. Therefore, DO saturation was relative constant and also no substrate consumption occurred in this phase (see Figure 1).

The lag phase was followed by the acceleration phase between 62 and 73 h, in which the growth rate gradually increased and biomass was formed. Then, the cells grew in the exponential phase at a constant, ideally maximum growth rate. The maximum growth rate was achieved between 73 and 89 h, where in the same time glucose concentration decreased exponentially. Afterwards, substrate limitation occurred, and growth rate slowed down between 89 and 109 h during the depletion of maltose concentration, before it reached stationary phase. When interpreting the biomass concentration; however, it must be taken into account that during fermentation a part of the biomass always attached to the reactor wall or to internals and therefore, falsified the biomass concentration in the samples taken. This applies in particular to the last samples in the time series.

In terms of initial aeration, 1 L/min (0.5 vvm) was found to be optimal in this study, as a higher initial aeration rate resulted in increased attachment of inoculated spores to the wall in the headspace of the bioreactor. Consequently, fewer spores are then present in the fermentation medium for germination. In addition, higher initial aeration was not necessary during the lag phase due to low metabolic activity. As described in Section 2.4, minimum DO saturation of the fermentation was maintained by controlling the flow of atmospheric air and by controlled addition of pure oxygen to the supply air. When the DO drops below 30%, the control starts and increases the atmospheric airflow up to 2.25 L/min (see Figure 1 at about 78 h). The air flow rate was limited to a maximum value of 2.25 L/min, as higher gassing rates resulted in increased accumulation of fungal mycelium in the headspace of the bioreactor. If necessary, pure oxygen was added to the supply air via the control system to ensure sufficient oxygen supply to the fungus (see the low flow of pure oxygen between about 70 and 95 h in Figure 1). At the end of the exponential phase, the air flow rate then dropped back to the initial value of 1 L/min.

From the pH and substrate courses in Figure 1, the metabolic activity of the fungus during the fermentation can be analyzed. The pH value started to drop approx. 55 h after inoculation, where the lag phase was ended and the acceleration phase was started. The uptake of the substrate, which is glucose during this period, by an electrochemical H^+^ gradient is probably responsible for the pH value to drop. The gradient was generated by H^+^ pumps in the plasma membrane, which pumped H^+^ out of the cell. This created an electrochemical gradient with a higher H^+^ concentration outside of the cell than inside, and therefore, the pH of the fermentation medium dropped [34]. The pH dropped until around 80% glucose was consumed. In the meantime, the breakdown of the disaccharide maltose, which is also present, produces two molecules of glucose. They would be afterwards also transported into the cell, which is shown by another decrease of pH value after around 89 h. Starting from 109 h, the amount of the substrate intake was not as high as in the exponential phase anymore; therefore, the pH value increased until the fermentation was ended.

### 3.3 Characteristics of pH courses and reproducibility in glass bioreactor

**FIGURE 2:**
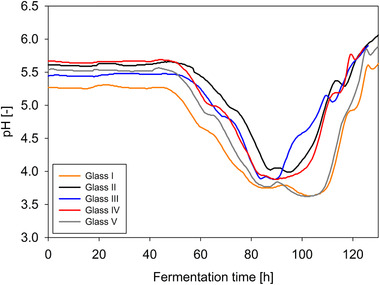
pH course of five fermentations of *Penicillium* sp. (IBWF 040‐09) in glass bioreactor (22°C, 350 rpm, initial aeration 1 L/min)

The pH value during the fermentation was only measured but not controlled, because it reflects the metabolic activity of the fungus and was, therefore, used to monitor the fermentations. The pH courses of five fermentations in glass bioreactor are compared in Figure 2 in order to examine its reproducibility and characteristics. In principle, the properties of the pH curves in the glass bioreactor are identical. The pH value initially drops while the organism takes up glucose. When the glucose is largely consumed, the pH value rises briefly. As the process continues, maltose is also metabolized, which is reflected in a further drop in the pH value. When both carbon sources are exhausted, the pH value rises again. Therefore, the fermentation of *Penicillium* sp. (IBWF 040‐09) in the glass bioreactor showed a good reproducibility.

It is also important to mention that between these five fermentations in the glass bioreactor under a complete identical condition, there could be seen a variation in time between 10 and 15 h of the peak trends, for example as the pH started to drop (between 45 and 55 h) and as the pH started to increase again (between 90 and 105 h). It can be observed that there are fermentations where the pH decreases later and increases sooner (i.e. narrow negative peak in pH value) and fermentations where the pH decreases sooner and increases later (i.e. broad negative peak in pH value). As described by Grimm et al. (2004) [35], there are two aggregation processes in the pellet formation of filamentous fungi. Each of these processes results in steady state condition which is shown in the stable relative particle concentration. Conidia bound to each other until reaching steady state in the first aggregation process. During the second aggregation step, germination, and hyphal growth occur, and the rest of the non‐germinated conidia will attach to the aggregates. The steady state of this aggregation step will lead to the growth of pellets.

The germination of the conidia of fermentations with narrow negative peak in pH course most probably occurred longer than in the other cases, which led to the longer time required for aggregation step until reaching the steady state. The more germinated conidia also led to more surface area available for second aggregation. In the same time, the exposed hyphae can also be detached from the aggregates due to the mechanical stress [36]. Presumably, these detached hyphae grew then into pellets [37, 38]. Thus, more pellets were generated and this led to a shorter time needed to consume all of the substrate. Consequently, the pH increased earlier. This assumption is also supported by the BDM data, where fermentations which show a narrow negative peak in pH value show a higher biomass (BDM glass II = 6.51 g/L, BDM glass III = 6.77 g/L) than fermentations which show a broader negative peak in pH value (BDM glass I = 5.26 g/L, BDM glass V = 5.44 g/L). The exact reason why these differences occurred between the fermentations which were carried out under identical conditions, cannot be clearly elucidated yet. The reproducibility of the determined biological parameters within the fermentations in the glass bioreactor, such as growth rate and yield coefficient, is further discussed in Sections 3.6 and 3.7.

### 3.4 Modifications of the SU bioreactor

The SU bioreactor is not equipped with an exhaust‐gas cooler because it was developed for cell culture and not for microorganism fermentation. One of the problems which arose during the fungi fermentation in the SU bioreactors was therefore the evaporation of the medium because the used inlet air and oxygen were dry gases. To minimize the evaporation, a humidifier was installed in front of the inlet‐air filter (between digital control unit and bioreactor) that can ensure that the air is saturated with water. A filter heater (UniVessel^®^ SU Filter Heater, Sartorius Stedim Biotech GmbH) was installed after the humidifier to ensure a sufficiently high temperature in the filter to avoid blockage due to possible condensation from the water‐saturated inlet air. Besides, the exhaust‐gas filter was constantly heated using another filter heater to avoid condensation from the water‐saturated exhaust gas.

**FIGURE 3:**
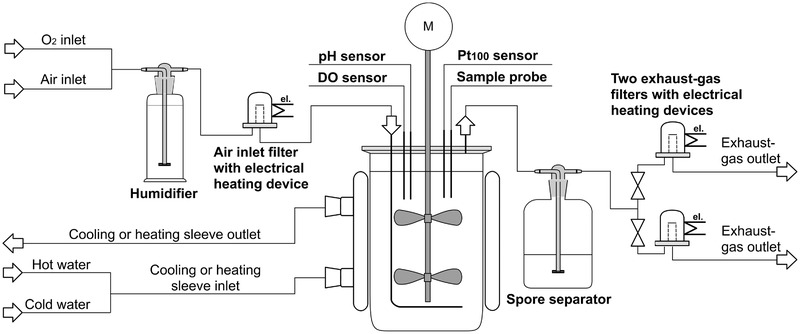
Experimental set‐up of modified single‐use bioreactor (figure created with [39])

The SU bioreactor was equipped with built‐in patches of DO and pH sensors. The measurement range of the pH sensor was between 6.0 and 8.0, while the pH value of fermentation processes in this study was between 3.5 and 6.0. Therefore, the built‐in pH sensor patch could not be used. Furthermore, the fungus overgrows both sensor surfaces during fermentation. This meant that the data from the DO sensor could not be used either. Therefore, standard pH (EasyFerm Plus PHI 325, Hamilton Company) and standard DO sensors (VisiFerm DO 325, Hamilton Company) were additionally inserted in the available PG 13.5 ports using compression fitting to adjust the correct height of the sensors in the bioreactor. The sensors were autoclaved separately and calibrated as described for the glass bioreactor (see Section 2.1). The installation of the electrodes in the SU bioreactor took place in a sterile workbench. A wash‐bottle filled with edible oil was also installed to avoid blocking of the exhaust‐gas filter due to spores. All fermentations of *Penicillium* sp. (IBWF 040‐09) in SU bioreactors in this study were carried out with these modifications (see Figure 3).

Since geometries and construction materials of the spargers in the two types of bioreactors differ (ring sparger in glass bioreactors made out of stainless steel, L‐sparger in SU bioreactors made out of polycarbonate), the volumetric mass transfer coefficient (k_L_a) for oxygen was measured according to [40] under the previously described fermentation conditions. In SU bioreactors a volumetric mass transfer coefficient of k_L_a = 14.4 ± 0.6 h^–1^ was determined. This was significantly lower than the k_L_a = 22.7 ± 0.3 h^–1^ determined in the glass bioreactor. The main reason for this is the varying size of the air bubbles, which is influenced by the geometry and, via the surface tension, also by the construction material of the sparger [41]. In the glass bioreactor the air bubbles are more uniform and above all smaller, so that a higher k_L_a value can be expected. However, due to the slow growth of the fungus this was no problem in all experiments, because same fungal growth could be observed in both bioreactor types (see Section 3.6). The shear stress depends not only on the stirrer tip speed but also on the aeration. Corresponding to a lower k_L_a value, a lower shear stress can also be expected in the SU bioreactors. This fact is more an advantage than a disadvantage, as it favors growth in pellet form.

### 3.5 Comparison of the pH courses in glass bioreactor and single‐use bioreactor

**FIGURE 4:**
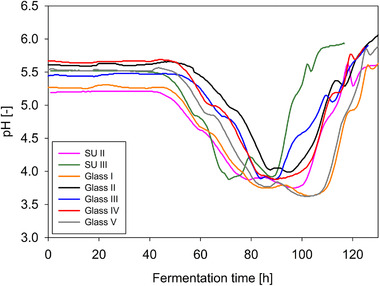
Comparison of pH courses of fermentations of *Penicillium* sp. (IBWF 040‐09) in SU and glass bioreactors under the same fermentation conditions (22°C, 350 rpm, initial aeration 1 L/min)

For the comparison of the fermentation in single‐use and glass bioreactors, the pH courses of fermentations in SU bioreactors are plotted in Figure 4 together with the fermentations in glass bioreactor. It can be seen that the metabolic activity of *Penicillium* sp. (IBWF 040‐09) during the fermentations are very similar in both bioreactor types. However, it must be noted that the point in time of the negative peak of the pH value curve varied more frequently in SU bioreactors. But the typical trends as previously described for fermentations in glass bioreactor (see Section 3.3) can also be seen in all fermentations in SU bioreactors. The lag phases of both fermentations in SU bioreactors showed here appear to be approx. 10 h shorter than in the glass bioreactor. The reason for the recognizable deviations between the fermentations can also be explained by the aggregation processes during pellet formation (see Section 3.3).

### 3.6 Biomass formation in glass bioreactor and single‐use bioreactor

**FIGURE 5:**
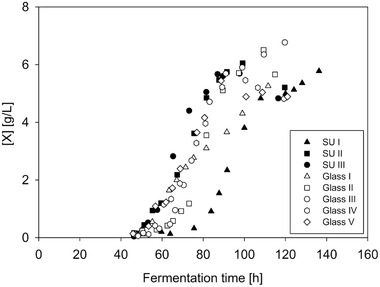
Comparison of the bio dry mass of fermentations of *Penicillium* sp. (IBWF 040‐09) in SU and glass bioreactors under the same fermentation conditions (22°C, 350 rpm, initial aeration 1 L/min)

As the aim of this study was to investigate the possibility to cultivate pellet‐forming fungi in SU bioreactors, one of the key parameters is the biomass concentration gained from the fermentations in SU bioreactors compared to glass bioreactor. In this study the biomass concentration in the fermentation medium was quantified with the bio dry mass (see Section 2.5). The comparison of BDM of fermentations in SU and glass bioreactors is shown in Figure 5. An increase in biomass could be determined after a cultivation time of approx. 45 h. The growth curves of the cultivations in SU bioreactors have similar courses as those in the glass bioreactor, except for a few fermentations in SU bioreactors, e.g. SU I in Figure 5. The slower biomass formation in fermentation SU I was in accordance to the delayed substrate consumption (see Figure 7).

The fermentations in SU bioreactors show greater variation in the determined biomass concentration, due to accumulation of biomass on the wall — mainly in the headspace of the bioreactor — as well as on the internals of the bioreactor. This could be seen within fermentations in SU bioreactors more often. With regard to all evaluable experiments; however, fermentations in both types of bioreactors have led to similar BDM when steady state was reached in the fermentations (BDM = 6.185 ± 0.707 g/L in SU bioreactors and BDM = 5.888 ± 0.697 g/L in glass bioreactors). In some cases, the measured BDM dropped in the last samples. This could be explained with the accumulation of the biomass on the bioreactor internals.

As could be observed during the experiments, the deviation of duplicates in the determination of the bio dry mass is significantly lower in the single‐use bioreactor than in the glass bioreactor. The reason for this is the different dimensioning of the respective sampling tube. While the sampling tube in the UniVessel^®^ Glass Bioreactor has an inner diameter of 4 mm, the corresponding tube in the UniVessel^®^ SU Bioreactor has an inner diameter of 5 mm. During sampling of suspensions with fungal pellets, larger differences occur with the smaller pipe diameter, since segregation takes place at the pipe inlet.

**FIGURE 6:**
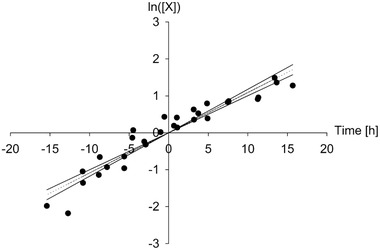
*t*‐Test statistical analysis of exponential growth phases of fermentations in glass bioreactors as explained in Section 2.9

However, *t*‐test statistical assessment of growth rate for five fermentations in glass bioreactors show good reproducibility, as depicted in Figure 6. A growth rate of μ = 0.1091 ± 0.0087 h^–1^ was determined for fermentation in the glass bioreactor with a prediction confidence of 95%. In the case of the SU bioreactor, a growth rate of μ = 0.0942 ± 0.0135 h^–1^ was determined. These values are comparable to the studies from Pirt and Righelato (1967) [42] and Righelato et al. (1967) [43] about chemostat cultures of *P. chrysogenum* in conventional glass bioreactors, which resulted in growth rates of μ = 0.086 h^‐1^ and 0.09 h^–1^, respectively. Another work reported lower growth rates, such as Adour et al. (2005) [17] with a growth rate of μ = 0.05 h^–1^ for *P. camembertii* in batch culture in a glass bioreactor.

Because both confidence intervals overlap strongly, it can be concluded that there are no differences in terms of growth rate in both bioreactor types. This result is also verified with Wilcoxon‐Mann‐Whitney test with a *P*‐value of *P* = 0.7857, which is greater than the threshold of *P* = 0.05. With this result, it can be concluded that the construction material polycarbonate — with its different surface properties — has no negative effect on fungal growth compared to glass and that the performance of the gassing system in the SU bioreactor is sufficient to supply the fungus despite the significantly lower k_L_a value.

### 3.7 Comparison of substrate consumption in glass bioreactor and single‐use bioreactor

**FIGURE 7:**
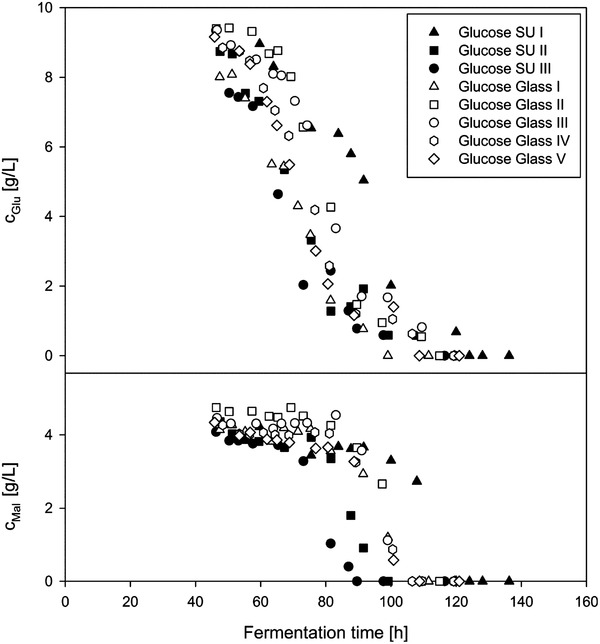
Comparison of glucose and maltose concentrations of fermentations of *Penicillium* sp. (IBWF 040‐09) in SU and glass bioreactors under the same fermentation conditions (22°C, 350 rpm, initial aeration 1 L/min)

From the substrate analysis (see Figure 7), it can be clearly seen that first of all glucose was consumed by the fungus because glucose is a monosaccharide and therefore, the fungus can directly transport it into the cell. In the case of maltose as substrate, extracellular enzyme is required to break down the bond between the two glucose molecules, which explains the preference of the fungus to first metabolize glucose.

The glucose decrease occurred in both bioreactor types in the same time course. Only fermentation SU I shows a delayed decrease of glucose. In the conducted experiments, such time delays could be observed several times, but all data reflect a good reproducibility. In addition, also the maltose concentration decreases in a similar way in both bioreactor types. According to the glucose trend, the maltose decrease of fermentation SU I was also delayed. This corresponds to the observed deviation in biomass formation (see Figure 5) and pH value (see Figure 4) of this fermentation.

Hille et. al. (2006) [30] described differences in pellet morphology between fermentations under the same conditions, except differences in spore batches. One resulted in fluffy structure on the edge of the pellets and solid in the middle, meanwhile other fermentations generated solid pellets outside but fluffy inside the pellets. Since the highest activity of the filamentous fungi is located at the peripheral area and the solid pellets contained more hyphae on the peripheral area than the fluffy ones, it can be concluded that the solid pellets have a higher conversion rate for substrates. Referring this observation to the result of the present study, it is possible that the pellets of the fermentation SU I were fluffy outside with a lower hyphae dense on the peripheral area. This could explain why the decrease of the substrates during fermentation SU I occurred later than the rest.

*t*‐Test statistical assessment of yield coefficient show good reproducibility in both bioreactor types. A yield coefficient of Y_X/S_ = 0.6199 ± 0.0623 g_X_/g_Glu_ was determined for fermentation in the glass bioreactor with a prediction confidence of 95% (see Section 2.9). In the case of the SU bioreactor, a yield coefficient of Y_X/S_ = 0.5520 ± 0.0875 g_X_/g_Glu_ was determined. Just as with the growth rates, there are no differences in the yield coefficients between the two reactor types. This result was confirmed by Wilcoxon‐Mann‐Whitney test which exhibits a *P*‐value of *P* = 0.5714, which is greater than the threshold of *P* = 0.05.

The yield coefficients obtained in this study are in good agreement with previous reports about the fermentation of *P. chrysogenum* using glucose as carbon source from van Gulik et al. (2001) [21], Christensen et al. (1995) [20], and Ryu and Hospodka (1980) [19] with 0.61 g_X_/g_Glu_, 0.51 g_X_/g_Glu_, and 0.45 g_X_/g_Glu_, respectively. A similar yield coefficient from the fermentation of the same species with sucrose as carbon source was observed by Mason and Righelato (1976) [18] with 0.48 g_X_/g_Suc_. Besides, Adour et al. (2005) [17] reported a yield coefficient of 0.59 g_X_/g_Glu_ from the fermentation of *P. camembertii* in 3 L glass bioreactor with glucose as carbon source and arginine as nitrogen source.

The substrate uptake rates (q_S_) of the fermentations in both bioreactor types were calculated from the growth rates and the yield coefficients (see Section 2.9) during the exponential growth phase in which glucose was consumed. Substrate uptake rates of q_S_ = 0.176 ± 0.029 g g^–1^ h^–1^ and q_S_ = 0.171 ± 0.044 g g^–1^ h^–1^ were determined within fermentations in glas bioreactor and SU bioreactors, respectively. Since substrate uptake rates are calculated from yield coefficients and growth rates (see Section 3.6), no statistical analysis is required to show that both bioreactor types lead to a similar fementation result. The maltose uptake rates were not calculated because the decrease in maltose concentration occurred to a higher extent only in the stationary phase.

### 3.8 Detection of the produced protease inhibitor

In preliminary studies, IBWF has tested the production of the protease inhibitior by *Penicillium* sp. (IBWF 040‐09) in conventional bioreactors. In order to confirm that this substance can also be produced using the modified SU bioreactor (see Figure 3), the mycelia from cultivations in SU bioreactors were harvested. After the extraction of mycelium and chromatographic analysis of the extracts, the fractions on a 96‐well‐plate were tested for protease inhibiting behavior of rhodesain (see Section 2.7 and 2.8).

Unfortunately, fractionation by HPLC did not result in good separation of the sample components and signal overlaps occurred to a greater extent in the chromatogram. Until now, it has not been possible to eliminate the strong peak overlap in HPLC. Therefore, the protease inhibitor could not be isolated or quantified so far. Protease inhibition activity in the fractions could nevertheless be detected using the method described in Section 2.8. In preliminary studies at IBWF, inhibition of up to 45% was observed in the HPLC fractions, although the fractions in question sometimes varied greatly from fermentation to fermentation (not shown). The relatively low inhibition was caused by the fact that the substance was spread in a broad region of wells (A5 to C6). In the future, further work needs to be done to improve HPLC fractionation and thus, to isolate the protease‐inhibiting substance in order to perform accurate quantification.

**FIGURE 8:**
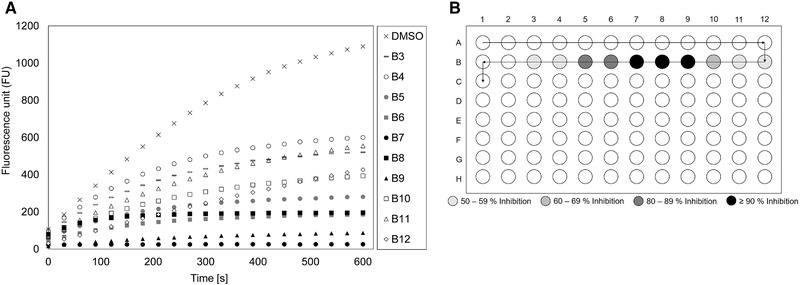
Fluorometric protease inhibition assay of the mycelium extract of *Penicillium* sp. (IBWF 040‐09) from fermentation SU V; (A) Results of the enzymatic assay (DMSO was used as a negative control without protease inhibitor); (B) Fractionation of the extract on a 96‐well‐plate (the arrow shows direction of the fractionation)

Therefore, in the present work, it can only be shown that the protease‐inhibiting substance was also formed in the SU bioreactors. Figure 8 shows an example of the inhibition assays performed on the extracts from the SU bioreactors. Three fractions (B7, B8, and B9) possess good inhibitory activity against the parasitic protease with over 90% inhibition, while several other fractions (B3‐B6 and B10‐B12) show a moderate protease inhibitory activity of more than 50%. The comparatively very high inhibitory effect of this particular sample could result from the fact that here the protease‐inhibiting substance is only present in a narrower well region than in the samples from the glass bioreactor.

As mentioned in the previous sections, there were always slight differences in the individual fermentations, although the fermentations were always carried out under the exact same cultivation conditions. This also resulted in variations in the activity of the protease inhibitor in each well in the enzyme assay from fermentation to fermentation. However, the results reveal that the protease‐inhibiting substance can also be produced in the SU bioreactor at least as well as in the glass bioreactor.

## 4 CONCLUDING REMARKS

This study has shown that the UniVessel^®^ SU Bioreactor from Sartorius Stedim Biotech GmbH was able to be used for filamentous fungi batch fermentations with some modifications required. Under the same cultivation conditions, the fermentations in SU bioreactor have resulted in the identical final biomass as in glass bioreactor within similar time frame (BDM = 6.185 ± 0.707 g/L in SU bioreactors and BDM = 5.888 ± 0.697 g/L in glass bioreactor). In addition, the pH courses in SU bioreactors compared to the pH courses in glass bioreactor also revealed an identical characteristic and therefore, it suggests the same metabolic activity of *Penicillium* sp. (IBWF 040‐09) during the fermentation in both bioreactor types.

Growth rates of μ = 0.0942 ± 0.0135 h^–1^ and μ = 0.1091 ± 0.00087 h^–1^ can be achieved from the fermentations in SU and glass bioreactors, respectively, which are in the range of other studies. The fermentations in SU and glass bioreactors resulted in yield coefficients of Y_X/S_ = 0.5520 ± 0.0875 g_X_/g_Glu_ and Y_X/S_ = 0.6199 ± 0.0623 g_X_/g_Glu_, respectively. These values are also within a range indicated by other works.

Based on the statistical analysis, growth rates, and yield coefficients of fermentations in SU and glass bioreactors are not significantly different. Thus, it can be concluded that the UniVessel^®^ SU can be used for the fermentation of filamentous fungi in pellet form despite the different construction material and the weaker gassing system in terms of volumetric mass transfer coefficient. Furthermore, it was shown that fungal fermentations can be carried out with good reproducibility in both the glass bioreactor and the SU bioreactor.

It was also possible to cultivate *Penicillium* sp. (IBWF 040‐09) in a way that a protease inhibiting substance was produced by the fungus. This substance could be separated from the fermentation broth via filtration and extraction of the pellets with a methanol/acetone mixture. The extract was divided in HPLC fractions (microwell fractions), at least three of which showed good protease inhibiting behavior. In further studies, the separation of the protease‐inhibiting substance by HPLC needs to be significantly improved to allow quantification.

## CONFLICT OF INTEREST

The authors have declared no conflict of interest.

## NOMENCLATURE

**Table:** 

c_Glu_	[g/L]	glucose concentration
c_Mal_	[g/L]	maltose concentration
k_L_a	[h^–1^]	volumetric mass transfer coefficient
q_S_	[g g^–1^ h^–1^]	substrate uptake rate
[S]	[g/L]	substrate concentration
t	[h]	time
[X]	[g/L]	biomass concentration
Y_X/S_	[g/g]	yield coefficient of biomass per substrate
*α*	[%]	confidence interval
μ	[h^–1^]	growth rate

## Supporting information

Supporting information.

## Data Availability

The data that support the findings of this study are available from the corresponding author upon reasonable request.
