# Supplementary material for: Applicability of a single‐use bioreactor compared to a glass bioreactor for the fermentation of filamentous fungi and evaluation of the reproducibility of growth in pellet form
Source: Eng Life Sci. 2021 Feb 25;21(5):324–39. doi: 10.1002/elsc.202000069 (PMC8092982; doi:10.1002/elsc.202000069)
Supplement: Supplementary file 1 — Supporting information. [file ELSC-21-324-s001.pdf]

Supporting Information

ITS sequence of *Penicillium* sp. (IBWF 040-09)
(ITS5/ITS4)

GAAGTAAAAGTAGTAACAAGG(ITS5)-
TTTCCGTAGGTGAACCTGCGGAAGGATCATTACCGAGCGAGGATTCTCTCGAAT
CCAACCTCCCACCCGTGTTTATTGTACCTTGTTGCTTCGGCGGGCCCGCCTCAC
GGCCGCCGGGGGGCATCTGCCCCCGGGCCCGCGCCCGCCGAAGACACCTTG
AACTCTGTATGAAAATTGCAGTCTGAGTCTAAATATAAATTATTTAAAACTTTCAA
CAACGGATCTCTTGGTTCCGGCATCGATGAAGAACGCAGCGAAATGCGATACGT
AATGTGAATTGCAGAATTCAGTGAATCATCGAGTCTTTGAACGCACATTGCGCC
CCCTGGTATTCCGGGGGGCATGCCTGTCCGAGCGTCATTGCTGCCCTCAAGCC
CGGCTTGTGTGTTGGGTCTCGTCCCCCTCCCCGGGGGGACGGCCCGAAAGG
CAGCGGCGGCACCGCGTCCGGTCCTCGAGCGTATGGGGCTTTGTCACCCGCT
CTGTAGGGCCGGCCGGCGCCTGCCGATCAACCACAATTTTTTTCCGGGTTGAC
CTCGGATCAGGTAGGGATACCCGCTGAACTTAA-
GCATATCAATAAGCGGAGGA(ITS4 RC)

ITS seq of *Penicillium* sp. (IBWF 040-09) shows:

99.47% identity to MH857606.1/ KP016835.1: *P. atrovenetum* strain CBS 243.56
99.47% identity to MH857605.1: *P. atrovenetum* strain CBS 241.56
99.65% identity to MH860682.1: *P. coralligerum* strain CBS 270.73
99.65% identity to KP016836.1: *P. coralligerum* strain CBS 114.69
99.29% identity to MH862703.1: *P. antarcticum* strain CBS 100492
99.82% identity to KP016829.1: *P. antarcticum* strain CBS 116939

(analyzed with standard nucleotide blast, given are the GenBank accession numbers, strain name, CBS number, all sequences contain small subunit ribosomal RNA gene, partial sequence; internal transcribed spacer 1, 5.8S ribosomal RNA gene, and internal transcribed spacer 2, complete sequence; and large subunit ribosomal RNA gene, partial sequence)
